# Plasma-Derived Polyreactive Secretory-Like IgA and IgM Opsonizing *Salmonella enterica* Typhimurium Reduces Invasion and Gut Tissue Inflammation through Agglutination

**DOI:** 10.3389/fimmu.2017.01043

**Published:** 2017-08-29

**Authors:** Gilles Bioley, Justine Monnerat, Marius Lötscher, Cédric Vonarburg, Adrian Zuercher, Blaise Corthésy

**Affiliations:** ^1^R&D Laboratory, Division of Immunology and Allergy, Lausanne University Hospital (CHUV), Lausanne, Switzerland; ^2^CSL Behring AG, Bern, Switzerland

**Keywords:** passive immunization, secretory IgA, secretory IgM, *Salmonella*, immune complexes

## Abstract

Due to the increasing emergence of antibiotic-resistant strains of enteropathogenic bacteria, development of alternative treatments to fight against gut infections is a major health issue. While vaccination requires that a proper combination of antigen, adjuvant, and delivery route is defined to elicit protective immunity at mucosae, oral delivery of directly active antibody preparations, referred to as passive immunization, sounds like a valuable alternative. Along the gut, the strategy suffers, however, from the difficulty to obtain sufficient amounts of antibodies with the appropriate specificity and molecular structure for mucosal delivery. Physiologically, at the antibody level, the protection of gastrointestinal mucosal surfaces against enteropathogens is principally mediated by secretory IgA and secretory IgM. We previously demonstrated that purified human plasma-derived IgA and IgM can be associated with secretory component to generate biologically active secretory-like IgA and IgM (SCIgA/M) that can protect epithelial cells from infection by *Shigella flexneri in vitro*. In this study, we aimed at evaluating the protective potential of these antibody preparations *in vivo*. We now establish that such polyreactive preparations bind efficiently to *Salmonella enterica* Typhimurium and trigger bacterial agglutination, as observed by laser scanning confocal microscopy. Upon delivery into a mouse ligated intestinal loop, SCIgA/M-mediated aggregates persist in the intestinal environment and limit the entry of bacteria into intestinal Peyer’s patches *via* immune exclusion. Moreover, oral administration to mice of immune complexes composed of *S*. Typhimurium and SCIgA/M reduces mucosal infection, systemic dissemination, and local inflammation. Altogether, our data provide valuable clues for the future appraisal of passive oral administration of polyreactive plasma-derived SCIgA/M to combat infection by a variety of enteropathogens.

## Introduction

Mucosal surfaces lining the gastrointestinal, the respiratory, and the genito-urinary tracts are the first port of entry for infectious agents, including bacteria, viruses, fungi, and parasites. At the mucosal surfaces, the predominant immunoglobulin is secretory IgA (SIgA), a complex made of polymeric IgA (pIgA), and bound secretory component (SC), which prevents interaction between pathogens and the target epithelium *via* immune exclusion, and limits subsequent tissue dissemination (1, 2). Although present in reduced abundance in mucosae, secretory IgM (SIgM) are believed to function similar to SIgA, as this is suggested from IgA-deficient patients exhibiting partially compensatory amounts of SIgM (3).

Passive immunization, the local or systemic administration of antigen-specific antibody molecules, represents a valuable intervention to achieve immediate protection against incoming invaders (4), well ahead of time-demanding activation of immune responses elicited by the infected host or deliberate vaccination. Mucosal delivery of preparations containing specific antibodies has allowed to demonstrate that passive immunization reduces infection in various species, including humans (5–8). However, this implies that sufficient amounts of antibody molecules are delivered in a biologically active form at the site of action. For gastrointestinal administration, this prerequisite is complicated by the need of delivering secretory immunoglobulins and not simply their polymeric counterpart. Indeed, association of polymeric immunoglobulins with SC drastically reduces the degradation of the antibodies exposed to protease-rich intestinal washes (9). Furthermore, proper anchoring of the antibodies in the mucus lining the epithelium requires that SC is present in the molecule (10, 11). Such biochemical features argue for an essential role for SC in SIgA function, which remains in need of characterization in a gastrointestinal model of infection. No data exist as to the role of (S)IgM in a similar environment.

Polyreactive secretory-like IgA (SCIgA) and secretory-like IgM (SCIgM) antibodies reconstituted from plasma-derived polymeric immunoglobulins and SC (12), when associated with the enteropathogen *Shigella flexneri*, have proven to delay infection of polarized epithelial cell monolayers used as a surrogate of the intestinal barrier (13). However, in the *in vivo* context, it has to be determined whether polyreactive plasma-derived SCIgA and SCIgM antibodies have the potential to counteract the damaging effect of enteropathogens. In this context, it is crucial that the biological activity, i.e., binding to the antigen, is maintained in the intestinal lumen and possibly beyond in the infected tissue. As no information on plasma-derived secretory immunoglobulins exists, we designed and performed several validation experiments in the context of immune complexes to demonstrate that such prerequisites for secretory immunoglobulins can be achieved. We have established that SCIgA, SCIgM, and secretory-like IgA and IgM (SCIgA/M) preparations bind to, and agglutinate, *Salmonella enterica* Typhimurium strain SL1344 (abbreviated St all along the paper). In the mouse intestinal lumen, such stable immune complexes keep most of the bacteria away from the target epithelium. After oral administration, this results in reduction of St epithelial entry and dissemination, as well as in decrease of production of inflammatory cytokines and recruitment of macrophages and neutrophils, all essential markers indicative of the control of infection.

## Materials and Methods

### Antibody Proteins Used in the Study

Human plasma-derived IgG preparations (Privigen, CSL Behring) were prepared as reported (14). Preparations containing pIgA and IgM antibodies were obtained from an ion-exchange chromatographic side fraction used in the large-scale manufacture of IgG from human plasma. The elution fraction containing IgA and IgM (IgA/M) at a 2:1 mass ratio was further processed to: (i) SCIgA/M, by combining *in vitro* IgA/M with recombinant human SC (10); (ii) pIgA at >50% (by mass), through removal of IgM from IgA/M by affinity chromatography specific for human IgM (CaptureSelect^®^ IgM, BAC B.V., Naarden, Netherlands); (iii) IgM, through removal of IgA from IgA/M by affinity chromatography specific for human IgA (CaptureSelect^®^ IgA). The secretory counterpart of pIgA and IgM was prepared by combination with recombinant human SC (10). Mouse monoclonal secretory IgASal4 (SIgASal4) specific for *S*. Typhimurium surface carbohydrates was prepared as described (15). Monoclonal antibodies and antisera used for ELISA, imaging, and flow cytometry are described in Table 1.

**Table 1:** Antibodies used in the study.

Antibody	Host	Provider	Clone	Label
Anti-human kappa chain	Goat	Pierce	Polyclonal	Biotin
Anti-human IgA1/2	Mouse	BD	G20-359	Biotin
Anti-human mu chain	Mouse	KPL	Polyclonal	Biotin
Anti-human gamma chain	Mouse	Sigma	Polyclonal	Biotin
Anti-human SC	Rabbit	Lab made	Polyclonal	–
Anti-rabbit IgG	Goat	Sigma	Polyclonal	HRP
Anti-rabbit IgG	Goat	Invitrogen	Polyclonal	AlexaFluor647
Anti-human SC	Rabbit	Lab made	Polyclonal	–
Streptavidin	–	Sigma	–	HRP
Streptavidin	–	GE Healthcare	–	Cy5
Anti-mouse CD16/32	Rat	BD	2.4G2	–
Anti-mouse CD3e	Hamster	BD	500A2	V500
Anti-mouse CD11b	Rat	BD	M1/70	PE*Cy7
Anti-mouse CD11c	Hamster	BD	HL3	FITC
Anti-mouse CD19	Rat	BD	1D3	APC-H7
Anti-mouse CD45	Rat	eBioscience	30-F11	AlexaFluor700
Anti-mouse Ly-6C	Rat	eBioscience	HK1.4	PE
Anti-mouse Ly-6G	Rat	eBioscience	1A8	APC
Anti-mouse F4/80	Rat	BioLegend	BM8	PerCP

### Bacterial Strain and Culture Conditions

The streptomycin-resistant virulent St (16) was used after growth to mid-log phase. The GFP-expressing St derivative was obtained in the laboratory.

### ELISA

To examine the biding capacity of human plasma antibodies to bacteria (13), 96 well plates (Maxi-Sorp, Nunc) were coated with 4 × 10^7^ cfu/well of St in PBS O/N at 4°C, prior to blocking with PBS-T containing 5% nonfat dry milk (NFDM). Serial dilutions of human plasma-derived monomeric IgA (mIgA) (9), pIgA, SIgA, IgM, SIgM, IgA/M, SCIgA/M, or IgG (starting at 0.5 µM) or SC (starting at 6.25 µM) were added. Bound antibodies were detected by incubation with biotinylated goat anti-human kappa chain (Pierce) or rabbit anti-human SC (17), followed by extravidin-horseradish peroxidase (HRP, Sigma) or goat anti-rabbit-HRP (Sigma), a in PBS-T containing 0.5% NFDM. The substrate for HRP was tetramethylbenzidine.

### Preparation of Immune Complexes

For administration to ligated intestinal loop and visualization of immune complexes, 2 × 10^6^ St were mixed with 100 µg of SIgASal4 or human plasma-derived pIgA, SIgA, IgM, SIgM, IgA/M, SCIgA/M, or IgG, or with 20 µg of SC in 100 µl PBS. For oral infection and agglutination efficiency, 2 × 10^7^ bacteria were mixed with either 40 µg, 200 µg, 1 mg, or 5 mg of human plasma-derived IgA/M or SCIgA/M in 150 µl PBS. The mixtures were incubated for 1 h at room temperature on a rotating wheel and, subsequently, used as such.

### Observation by Laser Scanning Confocal Microscopy of Immune Complexes

Immune complexes formed by St-GFP and plasma-derived antibodies were labeled in PBS-2% FCS with biotinylated mouse anti-human IgA1/IgA2 (BD Biosciences), biotinylated mouse anti-human mu chain (KPL), biotinylated mouse anti-human gamma chain (Sigma), or rabbit anti-human SC (lab made) for 1 h at room temperature. Detection was performed with cyanine 5 (Cy5)-conjugated streptavidin (GE Healthcare) or AlexaFluor647-conjugated goat anti-rabbit (Invitrogen) for 30 min at room temperature. After washing with PBS, labeled immune complexes were laid onto glass slides (Thermo Scientific), mounted with Vectashield reagent (Vector Laboratories), and visualized using a Leica SP5 confocal microscope equipped with a 63× objective. Images were processed with Imaris 8 software. To evaluate agglutination efficiency, the number of free St-GFP, the number of bacteria aggregates and their estimated size were determined on 10 different fields, in 5 independent experiments.

### Mice

Four week-old female Balb/c mice were obtained from Charles River Laboratories (L’Arbresle, France) and used at the age of 7–8 weeks. They were housed in the animal facility of the Lausanne University State Hospital under standard conditions. All experiments were approved by the State Veterinary Office, Lausanne, Switzerland (permit number VD2880) and carried out in accordance with the guidelines of the animal experimentation law (SR 455.163) of the Swiss Federal Government.

### Ligated Intestinal Loops

Mice starved overnight were injected intraperitoneally with 100 µl of anesthetics [10 mg/ml Ketasol-100 (Gräub) and 0.2% Rompun (Bayer) in PBS] per 10 g of body weight. The abdomen was incised, and the peritoneal lining was opened to expose the intestines. The ileum was ligated loosely to prevent ischemia and tissue damage at ~ 0.5 cm of each side of one Peyer’s patch (PP), using surgical thread (18). Two mice were used per experiment performed as follows: A 100-µl solution containing 2 × 10^6^ St alone (mouse 1) or as St-Ab immune complexes (mouse 2) was delivered into the luminal environment of the ligated intestinal loop using a 0.5 ml U-100 Insulin syringe (gauge 29G1/2; BD Biosciences). The intestine was then reintroduced into the abdomen, and the peritoneal cavity and skin around the initial incision were sewn back. Mice were sacrificed 1.5 h later, the PP was removed from the intestinal tissue and incubated 30 min in DMEM containing 2% FCS and 100 µg/ml gentamycin to kill extracellular bacteria. A PP upstream of the ligated loop was collected to serve as non-infected control. The bacterial load was determined as described (19). The fold decrease was calculated by dividing the CFUs/mg of the PP of the St alone animal by the CFUs/mg of the PP measured in the St-Ab mouse; for presentation of the data in Figure 4, the condition St alone was arbitrarily fixed at 1.

### Preparation of Tissue Sections and Observation by Laser Scanning Confocal Microscopy

Intestinal portions containing one PP were fixed in PBS-4% paraformaldehyde for 2 h at 4°C, with subsequent embedding in PBS-12% sucrose for 90 min at 4°C, followed by overnight incubation in PBS-18% sucrose at 4°C. Intestine portions were flushed into the lumen with optimal cutting tissue solution (Sakura Finetek, Torrance, CA, USA), followed by complete immersion. Sections cut at a 7-µm thickness were obtained, and blocking was carried out in PBS containing 5% mouse serum and 2% FCS (20). Bacteria were detected upon incubation in PBS-5% FCS-0.1% saponin with biotinylated IgASal4, followed by Cy5-labeled streptavidin (Amersham Biosciences). After washing, cell nuclei were stained with DAPI. Laser scanning confocal microscopy images were obtained with a Leica SP5 device in multi-track mode. Images were analyzed and processed with Imaris 8 software. All the images presented in the paper are representative of at least four sections and depict 3D reconstructions.

### Oral Infection of Mice and Bacterial Load Determination

Mice were orally infected with 2 × 10^7^ St alone or in complex with plasma-derived antibodies using a round tip stainless steel needle. Infected BALB/c mice were sacrificed 6 days post-infection. All visible PPs along the epithelium of the small intestine, the whole structure of MLNs and the spleen were collected. Bacterial loads were determined by distributing the equivalent of 15 mg of tissue lysate onto Luria-Bertani-agar plates supplemented with 90 µg/ml streptomycin (19).

### Assessment of Inflammation

Six days post-infection, mice were sacrificed, 4–6 PPs were collected per mouse and processed to cell suspensions (21). Cells (1.5 × 10^6^) were labeled with anti-CD16/32 monoclonal antibodies, followed by anti-CD45, -CD3, -CD19, -CD11b, -CD11c, -Ly-6C, -Ly-6G, -F4/80, and DAPI. Frequency of neutrophils (DAPI^−^CD45^+^CD3^−^CD19^−^autofluorescence^−^CD11b^+^CD11c^−^Ly-6C^−^F4/80^−^Ly-6G^+^) and macrophages (DAPI^−^CD45^+^CD3^−^CD19^−^autofluorescence^+^CD11b^+^CD11c^−^Ly-6C^−^F4/80^+^Ly-6G^−^) were recorded with a Gallios flow cytometer (Beckman Coulter). Alternatively, 1 × 10^6^ cells/well were seeded in round bottom 96-well culture plates and incubated for 24 h at 37°C. TNF-α and IL-6 secreted in the culture supernatants were quantified by ELISA (ELISA Max kits, BioLegend).

### Statistical Analysis

Statistical analysis was performed using Prism software (GraphPad Software, Inc., La Jolla, CA, USA). Bars represent median of each experimental group. The unpaired, non-parametric Mann–Whitney test was used to compare two experimental groups. The unpaired, non-parametric Kruskal–Wallis test, corrected with Dunn’s test for multiple comparisons, was applied to compare experimental groups. ns, non-significant; *, *p* < 0.05; **, *p* < 0.01; ***, *p* < 0.001; ****, *p* < 0.0001.

## Results

### Plasma-Derived Antibodies Interact with St

**Figure 1:**
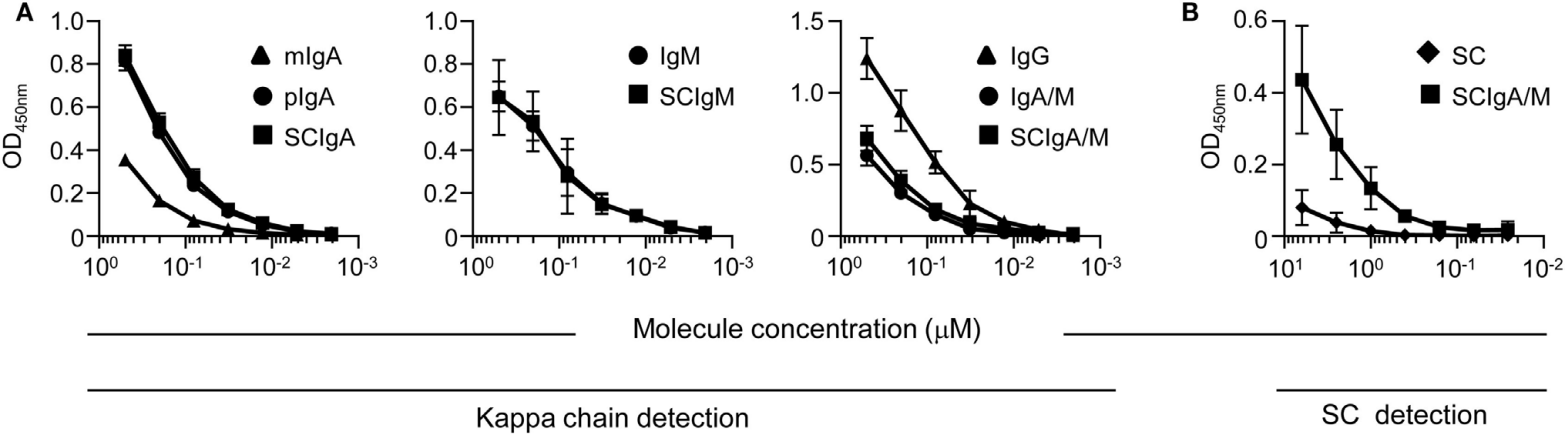
Plasma-derived antibody formulations interact with *Salmonella enterica* Typhimurium strain SL1344 Binding of equimolar concentrations of **(A)** plasma-derived antibodies or **(B)** recombinant secretory component (SC) or secretory-like IgA and IgM (SCIgA/M) to coated *Salmonella enterica* Typhimurium strain SL1344 as determined by ELISA. Data are the compilation of four independent experiments performed in duplicates and are depicted as means ± SD.

The ability of different molecular forms of plasma-derived IgA and IgM, or IgG, or recombinant SC to interact with St was first tested by ELISA. All antibody preparations displayed a dose-dependent recognition of the bacteria, yet at different levels (Figure 1A). The binding capacity of mIgA to St was lower than that of pIgA or SCIgA, consistent with increased avidity of the latter two. The same held true for IgM, SCIgM, IgA/M, and SCIgA/M preparations, which all demonstrated a potent capacity to interact with the bacterium. Somewhat surprisingly, the strongest signal was obtained with IgG, suggesting a substantial St-specific humoral response in the plasma of donors (22, 23). Consistent with a similar binding capacity of polymeric and secretory forms of IgA, IgM, and IgA/M, SC demonstrated a very limited ability to interact with St (Figure 1B).

### Plasma-Derived IgA/M Preparations Mediate St Agglutination

**Figure 2:**
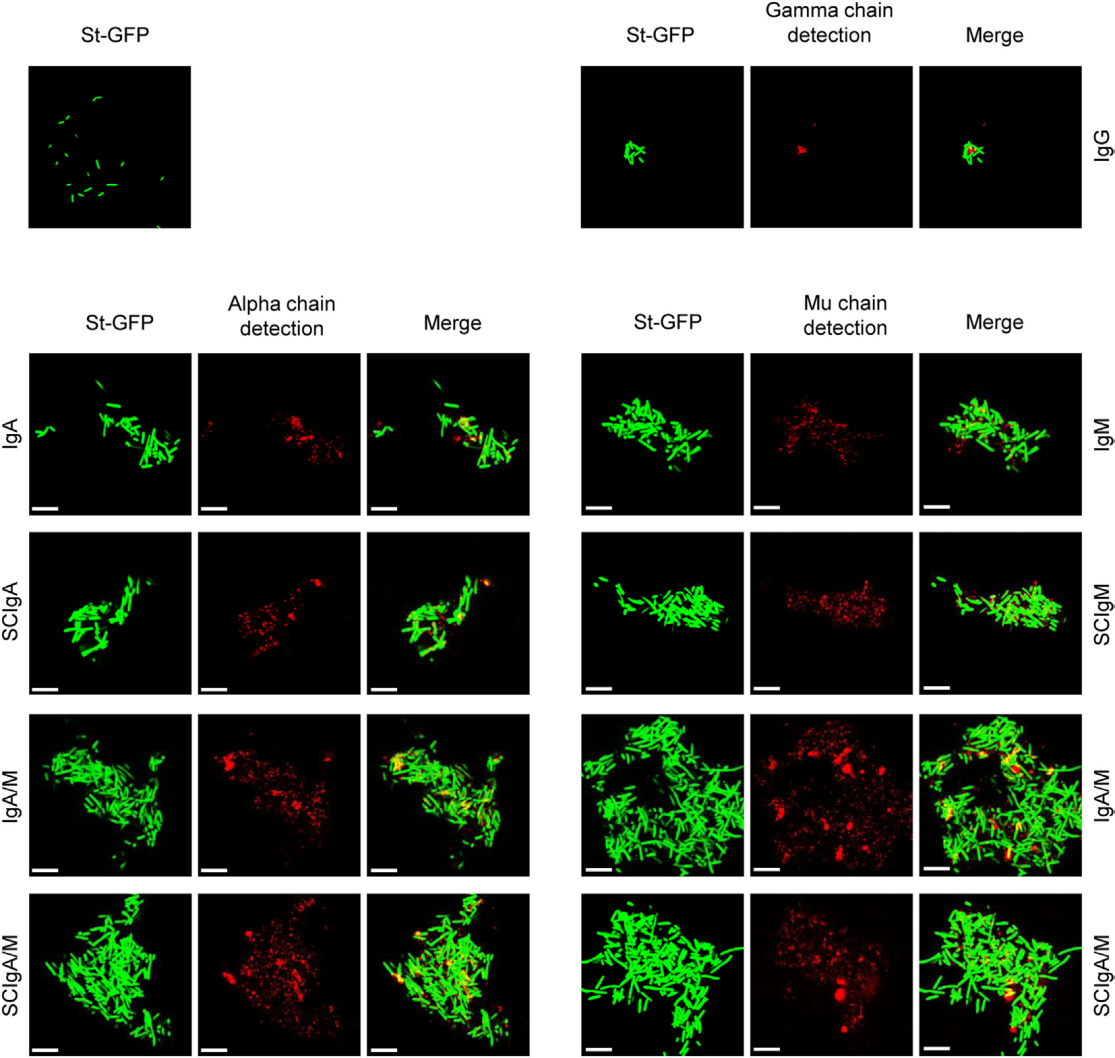
Association of plasma-derived antibody formulations with *Salmonella enterica* Typhimurium strain SL1344 (St) promotes agglutination. Laser scanning confocal microscopy images of immune complexes of 2 × 10^6^ St associated with plasma-derived antibodies. Bacteria constitutively expressing GFP appear in green. Bound IgA, IgM, or IgG were detected with biotinylated antibodies directed against the alpha, mu, or gamma chain, followed by streptavidin conjugated to cyanine 5, yielding red signals after image processing. Images are representative of one observed field obtained from 5 to 10 observations from three independent slides. Scale bars: 10 µm.

To further characterize the interaction of plasma-derived antibodies with St, we next prepared immune complexes in solution by mixing St-GFP with plasma-derived antibodies or recombinant SC. pIgA, SCIgA, IgM, and SCIgM antibodies all demonstrated the capacity to promote agglutination (Figure 2), whereas in the absence of antibodies, only isolated bacteria were observed. The formation of aggregates was due to the presence of antibodies all over the immune complexes, as detected with specific anti-alpha and -mu chain antibodies. Despite the high ability of IgG to interact with bacteria (Figure 1), only tiny and rare aggregates formed, presumably due to the nature of the antibody carrying one F(ab′)_2_ only. The highest degree of bacterial agglutination was observed with IgA/M and SCIgA/M, with contribution of both IgA and IgM, as demonstrated by alpha- and mu-chain-derived signals in the immune complexes. As anticipated, the limited or lacking interaction between St and mIgA or SC resulted in the absence of bacteria agglutination (data not shown).

**Figure 3:**
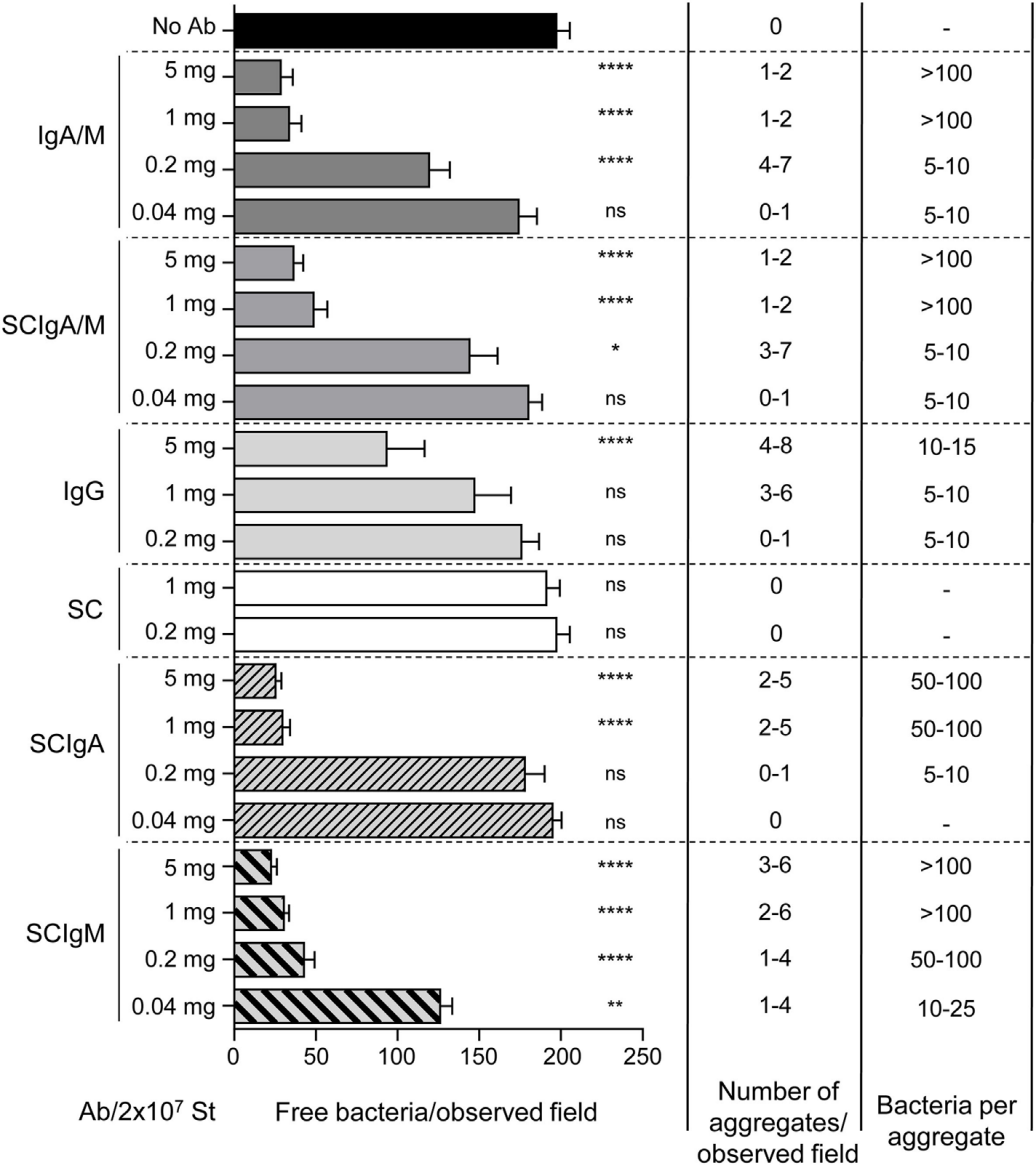
Quantification of the agglutination property of IgA and IgM (IgA/M), secretory-like IgA and IgM (SCIgA/M), IgG antibodies or secretory component (SC). 2 × 10^7^ *Salmonella enterica* Typhimurium strain SL1344 (St) alone or in combination with decreasing amounts of protein formulations was observed by laser scanning confocal microscopy. Using the Imaris 8 software, parameters, including the number of free fluorescent bacteria, the number of aggregates, and the estimated number of bacteria per aggregate were evaluated on 10 different fields from one experiment repeated 5 times. Numbers are means ± SD. Statistical analyses were performed by comparison with the “No Ab” experimental group. Ab, antibody.

The agglutination capacity was quantitated by counting free (i.e., not contained in aggregates) St-GFP and immune complexes prepared at different antibody/SC to bacteria ratios. About 200 free St-GFP were counted by observation field in the absence of antibodies (Figure 3). A small number of huge bacterial aggregates, containing more than 100 bacteria, were observed when immune complexes were prepared with 5 or 1 mg of IgA/M or SCIgA/M, resulting in only 20% of free St-GFP as compared to the control condition. The efficiency of agglutination diminished in the presence of 0.2 mg IgA/M or SCIgA/M, as reflected by the smaller size of formed aggregates. In the presence of 0.04 mg antibodies, complexes were barely observed. In the presence of IgG, only small bacteria aggregates were detected and at least five times more antibody were necessary to end up with the same number of free St-GFP when compared to IgA/M or SCIgA/M (Figure 3). No complexes were found when SC was mixed with St.

These data demonstrate that the interaction of (SC)IgA and (SC)IgM with St translates into the formation of bacterial aggregates, an essential prerequisite for the prevention of pathogen entry into mucosal tissues.

### Plasma-Derived IgA/M and SCIgA/M Preparations Limit St Entry into PPs

**Figure 4:**
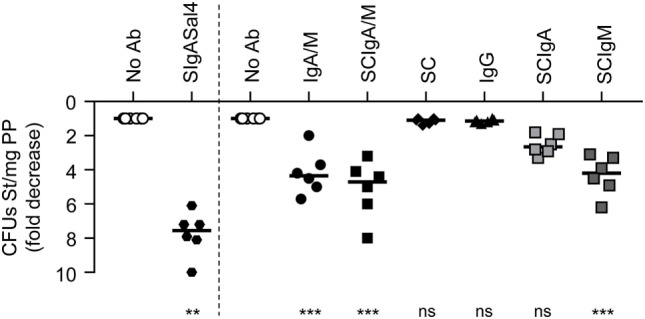
Reduction of *Salmonella enterica* Typhimurium strain SL1344 (St) entry into Peyer’s patches (PPs) by antibody preparations following delivery in a ligated intestinal loop. 2 × 10^6^ St was administered alone or in the presence of IgA and IgM (IgA/M), secretory-like IgA and IgM (SCIgA/M), secretory component (SC), IgG, SCIgA, SCIgM, and SIgASal4 as a positive control into a ligated intestinal loop containing a PP. At 1.5 h, PPs were collected, lysed, and bacterial counts were determined by plating. Data are presented as fold decrease with respect to the “No Ab” experimental group (see Materials and Methods). Bars represent medians. Ab, antibody; CFUs, colony forming units.

To test whether IgA/M and SCIgA/M can indeed mediate immune exclusion and prevent St entry into PPs, we administered immune complexes formed with 100 µg antibody into ligated intestinal loops containing one PP. St-specific SIgASal4 monoclonal antibody protects mice when given as backpack, an approach that releases the antibody in the circulation and leads to the transport of J chain-containing IgA into secretions (24). When delivered in the form of immune complexes with St, SIgASal4 was able to significantly reduce the entry of bacteria in PPs (Figure 4, left). Consistent with their ability to mediate St agglutination, a significant decrease in bacterial load was also observed in PPs after injection of immune complexes formed with IgA/M and SCIgA/M, as compared to St alone (Figure 4). No protective effect of either IgG or SC was detected. When IgA and IgM purified from IgA/M preparation and reconstituted in secretory-like antibodies were tested, both demonstrated the ability to reduce bacterial load in PPs, with superior performance of SCIgM (Figure 4). This may be explained either by the decameric valency of IgM, or the presence of some mIgA antibodies in the pIgA preparation, which most likely did not participate in limiting interaction with the intestinal PP.

**Figure 5:**
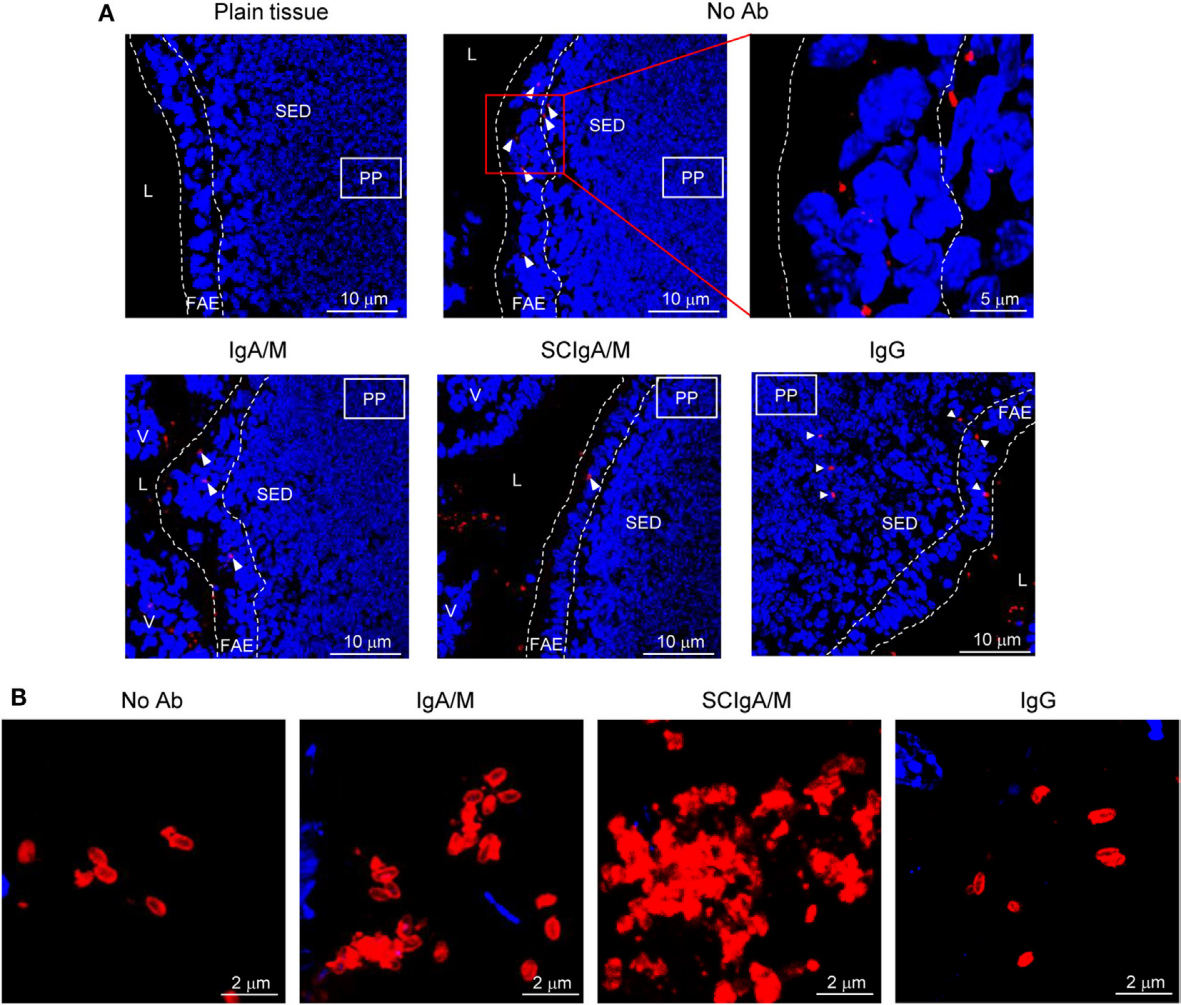
Agglutinating antibodies limit diffusion of St from the lumen to the underlying tissue. **(A)** Visualization by laser scanning confocal microscopy of the fate of 2 × 10^6^ St administered alone or in complex with IgA/M, SCIgA/M, or IgG in a ligated intestinal loop containing a PP. The upper left panel shows the uninfected tissue for comparison. St is displayed in red, and cells appear in blue (DAPI). One representative image out of 20 is depicted for each antibody tested. L, lumen; V, villus; FAE, follicular-associated epithelium; SED, subepithelial dome region. **(B)** High magnification of the lumenal space within a ligated intestinal loop administered with St alone or in complex with SCIgA/M, IgA/M, or IgG. Ab, antibody.

Laser scanning confocal microscopy visualization of PP sections showed that St administered alone in a ligated intestinal loop is found within the follicular-associated epithelium and at the interface with the subepithelial dome region 1.5 h after injection (Figure 5A; arrowheads). Reduced numbers of St were observed within the follicular-associated epithelium when SCIgA/M-based immune complexes were injected, a feature also observed with IgA/M, yet to a lesser extent (Figure 5A). Occasional bacteria were detected in the subepithelial dome region, while many remained detectable in the lumen environment, suggesting operative mucosal immune exclusion. Consistent with the data of bacterial agglutination (Figure 3), association with IgG yielded similar distribution as St alone. In the presence of SCIgA/M or IgA/M, persistence of bacterial aggregates in the lumen, justifying of limited entry, was confirmed by specific staining of St (Figure 5B). Only single or pairs of bacteria were observed in the absence of antibody or in the presence of IgG (Figure 5B). The sum of the data validates plasma-derived SCIgA/M and IgA/M in their function of limiting mucosal entry of St and demonstrates the maintenance of stable immune complexes essential to ensure protective immune exclusion. We next sought to visualize the impact of the antibodies on the dissemination of St. Because data in Figures 1–4 demonstrated the functionality of IgA/M and SCIgA/M in *in vitro* and *ex vivo* assays and that the two antibodies are naturally found in secretions, we used these two molecular forms for oral administration.

### Plasma-Derived SCIgA/M Limits St Infection and Dissemination after Oral Administration

**Figure 6:**
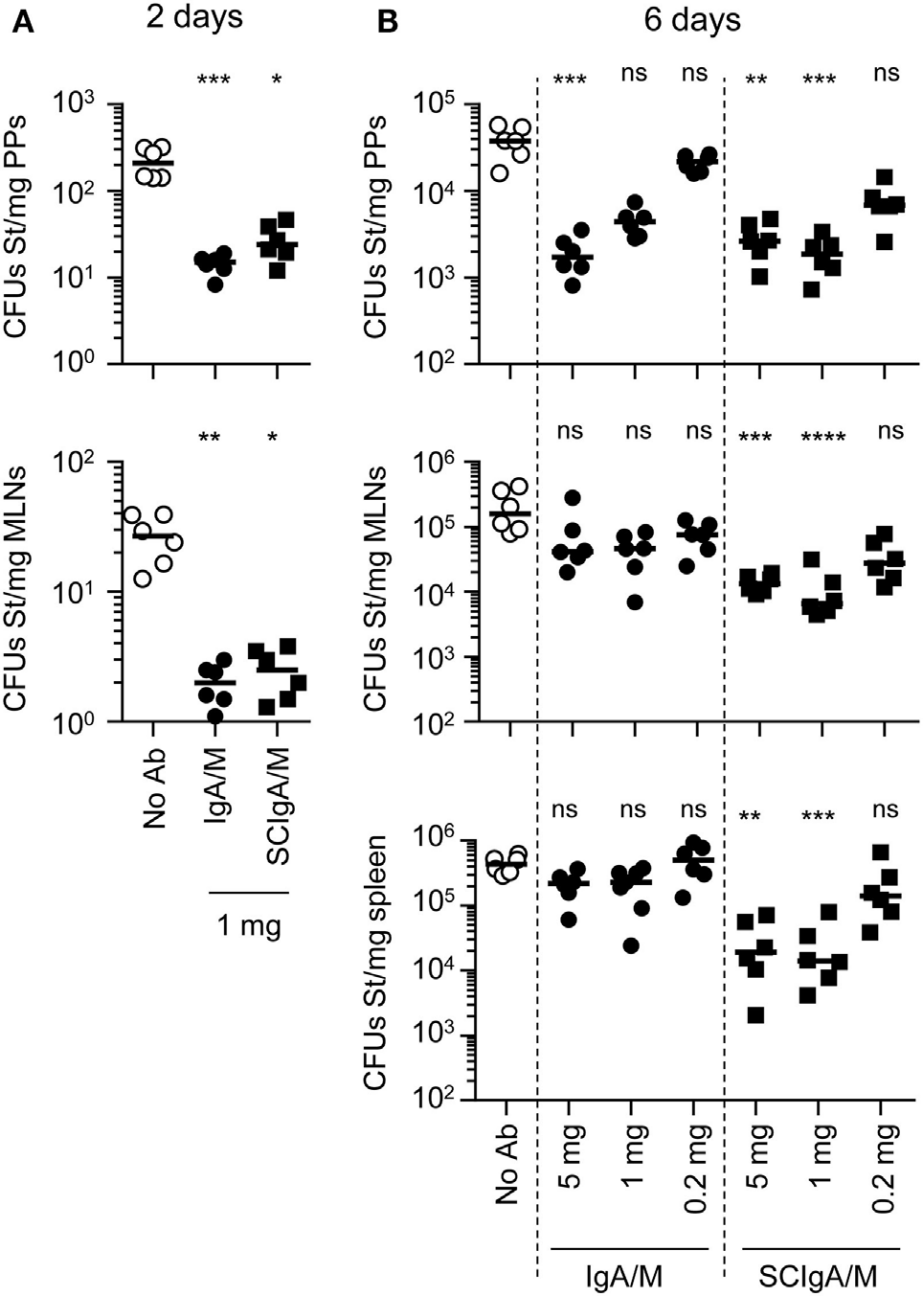
Limitation of *Salmonella enterica* Typhimurium strain SL1344 (St) dissemination in the presence of antibody preparations after oral administration. Mice were orally administered 2 × 10^7^ St alone or in association with preparations IgA and IgM (IgA/M), secretory-like IgA and IgM (SCIgA/M), or IgG. **(A)** Bacterial counts in mucosal tissues [Peyer’s patches (PPs), mesenteric lymph nodes] were determined by plating of tissue lysates 48 h post-delivery of St or immune complexes prepared in the presence of 1 mg antibodies. **(B)** Similar experiment as in **(A)**, using immune complexes made of 2 × 10^7^ St and decreasing amounts of antibodies, with bacterial counts assessed in PPs, mesenteric lymph nodes and the spleen at day 6 post-administration. Experiments were performed twice, with three mice per group, and the compilation of the two experimental sets is depicted. Bars represent medians. Statistical analyses were performed by comparison with the “No Ab” experimental group. Ab, antibody; MLNs, mesenteric lymph nodes; CFUs, colony forming units.

Having demonstrated that IgA/M and SCIgA/M antibodies bound to St limit its diffusion from the lumen into PPs, we sought to evaluate whether biological activity was preserved after oral administration. When combined with 1 mg of IgA/M or SCIgA/M, the number of St recovered from PPs and mesenteric lymph nodes 2 days post-infection was significantly reduced as compared to the administration of the bacteria alone (Figure 6A). When the infection was left to develop for 6 days, analysis of mice post-infection revealed that SCIgA/M protected mice better than IgA/M, especially with respect to dissemination beyond PPs. Increasing the amount of antibody to 5 mg did not improve the performance of SCIgA/M, which displayed the same protective effect when the bacterial load was measured in PPs, mesenteric lymph nodes, and the spleen (Figure 6B). By contrast, IgA/M turned out to be effective in reducing the bacterial load in PPs only. Despite a loss in statistical significance, bringing down the amount of SCIgA/M to 0.2 mg in the immune complex still allowed to lower bacterial dissemination.

### Plasma-Derived SCIgA/M Quenches Inflammatory Circuits Induced by St Infection

**Figure 7:**
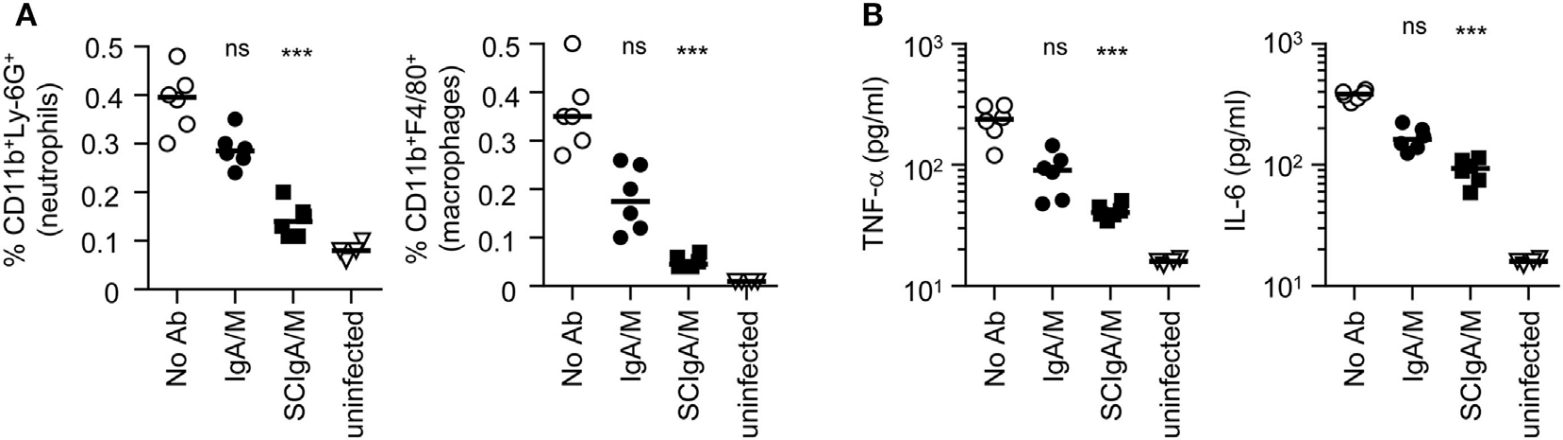
Reduced markers of inflammation after oral delivery of *Salmonella enterica* Typhimurium strain SL1344 (St) in the presence of antibody preparations. **(A)** Frequency of neutrophils and macrophages was determined as a function of the total number of cells in PPs, 6 days post-administration of 2 × 10^7^ St alone or in association with 1 mg of IgA/M or SCIgA/M preparations. **(B)** Pro-inflammatory cytokine secretion measured in cell culture suspensions of PPs from the same experimental groups as in **(A)**. Experiments were performed twice, with three mice per group, and the compilation of the two experimental sets is depicted. Bars represent medians. Statistical analyses were performed by comparison with the “No Ab” experimental group. Ab, antibody.

Secretory IgA has been demonstrated to dampen inflammatory processes in the gastrointestinal tract, which is considered beneficial for the host by preventing tissue destruction in pathological situations. After oral infection with St, rapid gastrointestinal inflammation is triggered; relevant markers include an increase in frequencies of neutrophils and macrophages, as well as elevated production of TNF-α and IL-6 (Figures 7A,B; compare uninfected with “No Ab” experimental conditions). When SCIgA/M-based immune complexes were administered, a reduction in both phagocyte recruitment and pro-inflammatory cytokine production were measured in PPs at day 6 (Figures 7A,B). The effect was less pronounced in presence of IgA/M. The data suggest that keeping St away from the gastrointestinal epithelium ensures protection in a context which does not need to rely on exacerbated inflammatory responses (25).

## Discussion

This study was designed to examine several unknowns associated with the fate and consequence of orally delivered plasma-derived immunoglobulins, including SCIgA, SCIgM, and a mixture of the two, referred to as SCIgA/M, in a mouse model of *S*. Typhimurium infection. We found that all preparations interact with the bacterium, resulting in the formation of immune complexes of increasing sizes as a function of the antibody isotype/valency. Immune complexes administered orally reduced infection of PPs, the privileged site of entry of *Salmonella* (26), in a hierarchy-dependent manner, with SCIgA/M exhibiting the highest effect. Stable immune complexes persist in the intestinal lumen, triggering limited production of inflammatory cytokines in comparison with the “No Ab” scenario. The sum of the data indicates that immune exclusion, which secludes bacteria from contact with mucosal membranes, is also operative with polyreactive plasma-derived secretory-like immunoglobulins in the gastrointestinal tract. The data provide valuable clues as to the optimal molecular form to be used to prevent bacterial dissemination and to limit local inflammation, two beneficial features associated with passive immunization.

It is known that SC contributes to important structural features of SIgA, including stability and mucosal location (11). Our results further stress that the secretory counterpart of the best performing antibody, i.e., SCIgA/M, represents the most optimal molecular preparation to be used *in vivo*. Importantly, biologically active human SC can be produced in Chinese hamster ovary cells, while polyreactive, plasma-derived polymeric immunoglobulins can be fractionated in large amounts when manufacturing other plasma-derived products. We show that such preparations are able to agglutinate highly invasive St to thus limit its entry when given in the form of immune complexes in the murine gastrointestinal tract. Remarkably, even a low percentage in polyreactive preparations of antibodies specific for a given pathogen seems to be sufficient to limit diffusion in target gut tissues.

The approach represents a sound alternative to recombinant technologies (27–31), which in the case of IgA and IgM, may suffer from the complication that a plethora of molecular forms, including monomers and partially assembled polymers, reduces the yield of useful molecules for the final assembly into secretory immunoglobulins. The fact that administration of St-SCIgA/M complexes brings down markers of infection and inflammation argues in favor of the maintenance of the antibody structure and activity in the conditions of administration chosen, i.e., a phosphate-buffered saline solution. Improvements, including neutralization of gastric acidity prior to antibody oral delivery and/or encapsulation in formulations protecting the antibody from the action of intestinal proteases may deserve consideration in future developments. The latter approach may be worth exploring while keeping in mind that the high molecular weight of secretory antibodies may represent a technical bottleneck and that their intrinsic stability may be well sufficient to guarantee efficacy.

We show that the contribution of both the IgA and IgM isotypes can be underscored in the structure–function relationship of the SCIgA/M preparation. SIgA has been assigned the role of chief antibody at mucosal surface, and much is known on how it controls and regulates local homeostasis vis-a-vis pathogens and commensals (32, 33). High-affinity, inducible SIgA are involved in response against pathogens, with a mode of action relying on blocking the interaction between antigens and the epithelia overlying mucosae. On the other hand, polyreactive IgM that exist without known antigenic exposure or deliberate vaccination are referred to as “natural,” whereas IgM generated in response to defined antigen stimulation display fairly high specificities (34). The relevance of natural IgM in controlling infections was first seen in primary immunoglobulin-deficient patients highly susceptible to recurrent bacterial, viral, fungal, and parasitic infections. Immunodeficient animals have their capacity to control certain infections restored after infusion with natural IgM (35, 36). It has been proposed that at mucosal surfaces, pentameric IgM transported by the epithelial polymeric immunoglobulin receptor contributes to immune exclusion by direct pathogen recognition (37), thus limiting passage across the epithelial barrier. Our data show that plasma-derived reconstituted SCIgM can fulfill this function when combined with St. In support of the role of either isotype in mucosal defense, the SCIgA/M preparation comprising the two isotypes yielded the best protective activity. Moreover, the importance of using secretory immunoglobulins in protection, including SIgM, has been identified through mice lacking the polymeric immunoglobulin receptor (38).

Although our work focused on a particular enteropathogen in the gut context, one can envisage other applications targeting different microorganisms in mucosal environments comprising the genito-urinary and the respiratory tracts. Delivery of monoclonal antibodies *via* the airways has shown promising potential to treat lung tumors (39). Because the environment of the respiratory tract is not endowed with as much proteases as in the gut, it is expected that the stability of the administered antibody is augmented. In comparison with the gastrointestinal tract, the low number of bacteria present in the conducting airways will result in less possible competition for the antibody delivered exogenously. Reduced digestion and improved availability are two features that suggest that less antibody molecules may be needed for passive immunization in the lung tissue.

In contrast to classical vaccination, an interesting feature of passive immunization is that it is based on direct administration of bioactive molecules, and, therefore, allows facilitated translation from an animal experimental model to future human application. Indeed, in the context of active vaccination, compartmentalization of the mucosal immune system may prevent conclusive interpretation from interspecies experiments, on the one hand, and complicate the identification of the best route of administration to ensure robust local immune responses (40). In the face for the need for therapeutic interventions targeting mucosal pathogens [*Clostridia*, HIV, *Helicobacter pylori*, respiratory syncytial virus, *Chlamydia, Pneumococcae, Giardia* (41)] for which no vaccine exist, it sounds reasonable to envisage that passive immunization with plasma-derived secretory immunoglobulins operative at mucosal surfaces offers an interesting and valuable alternative.

An alternative or an addition to already existing efficient vaccines (42–44), passive immunization can deliver protective levels of antibodies directly to the susceptible mucosal site where most infections begin. Moreover, passive mucosal administration of secretory antibodies with a large spectrum of specificity might overcome issues related to antibiotic resistance. On the top of that, it presents the advantage of being immediately operative in a sick organism (4, 5), and serve as a short-term protection before vaccine-induced immunity is achieved in high-risk populations. Another obvious advantage lies in the intrinsic capability of the antibody to neutralize the pathogen at a very early stage of infection, or ideally prior to the initiation of the infection. Passive protection by transfer of antibody from maternal origin represents in many mammals including humans an efficacious and natural mechanism to prevent mucosal infection in newborns lacking a fully matured immune system (45, 46). In this respect, the amount of antibody administered was set to remain close to the scenario that takes place naturally during breastfeeding; human colostrum contains 12 mg/ml SIgA and milk 1.5 mg/ml (47). Finally, passive immunization can overcome the intrinsic difficulty to elicit mucosal immune responses in the pro-tolerogenic intestinal environment (48, 49).

The dissection of the mode of action of plasma-derived SCIgA/M when orally delivered in the form of immune complexes, together with the demonstration that they maintain their biological activity in the harsh gut environment, opens the way to the future exploration of their potential in prophylactic and possibly therapeutic applications against a large array of mucosal pathogens. Such future developments may help to warrant immediate intervention procedures in a large population of at-risk individuals, to circumvent the need for priming/activation of the immune system, and as importantly, may participate to reduce the danger of antibiotic overuse.

## Ethics Statement

All experiments were approved by the State Veterinary Office, Lausanne, Switzerland (permit number VD2880) and carried out in accordance with the guidelines of the animal experimentation law (SR 455.163) of the Swiss Federal Government.

## Author Contributions

GB, CV, AZ, and BC designed the experiments. GB and JM performed research and analyzed data. ML provided reagents. GB, CV, and BC wrote the manuscript. All authors have read, critically revised, and approved the final manuscript.

## Conflict of Interest Statement

The authors declare that the research was conducted in the absence of any commercial or financial relationships that could be construed as a potential conflict of interest.

## Abbreviations

mIgA, monomeric IgA; pIgA, polymeric IgA; PP, Peyer’s patch; SC, secretory component; SCIgA, secretory-like IgA; SCIgM, secretory-like IgM; SIgA, secretory IgA; SIgM, secretory IgM; St, *Salmonella enterica* Typhimurium strain SL1344.
